# Supplementary material for: O-GlcNAcylation promotes the cytosolic localization of the m6A reader YTHDF1 and colorectal cancer tumorigenesis
Source: J Biol Chem. 2023 Apr 21;299(6):104738. doi: 10.1016/j.jbc.2023.104738 (PMC10208891; doi:10.1016/j.jbc.2023.104738)
Supplement: Supplementary Table [file mmc1.docx]

**Supplementary Table 1 YTHDF1-interacting importins and exportins identified in the MS analysis**

| Protein identified | PSM^a^ count | Coverage |
| --- | --- | --- |
| IPO5, Importin-5 | 13 | 0.16 |
| IMA1, Importin subunit alpha-1 | 10 | 0.321 |
| IMB1, Importin subunit beta-1 | 9 | 0.142 |
| IPO9, Importin-9 | 6 | 0.125 |
| IPO4, Importin-4 | 5 | 0.06 |
| IMA7, Importin subunit alpha-7 | 4 | 0.099 |
| IMA6, Importin subunit alpha-6 | 3 | 0.065 |
| IMA5, Importin subunit alpha-5 | 2 | 0.026 |
| XPO2, Exportin-2 | 6 | 0.123 |
| XPO1, Exportin-1 | 2 | 0.024 |

^a^, PSM: the peptide-spectrum matches
